# Association between Statins Types with Incidence of Liver Cancer: An Updated Meta-analysis

**DOI:** 10.2174/0929867330666230701000400

**Published:** 2023-10-03

**Authors:** Xingfen Zhang, Dandi Lou, Rongrong Fu, Feng Wu, Dingcheng Zheng, Xueqiang Ma

**Affiliations:** 1 Department of Liver Disease, Ningbo No. 2 Hospital, Ningbo, Zhejiang, China;; 2 Key Laboratory of Diagnosis and Treatment of Digestive System Tumors of Zhejiang Province, Ningbo No. 2 Hospital, Ningbo, Zhejiang, China;; 3 The First Clinical Medical College, Zhejiang Chinese Medical University, Hangzhou, Zhejiang, China;; 4 Department of General Surgery, Ningbo No. 2 Hospital, Ningbo, Zhejiang, China;; 5 Department of Hepatobiliary Surgery, Zhuji People's Hospital, Shaoxing, Zhejiang, China

**Keywords:** Liver cancer, statin, lipophilic statins, hydrophilic statins, incidence, meta-analysis

## Abstract

**Background:**

Previous studies have found a potential role for statins in liver cancer prevention.

**Objective:**

This study aimed to explore the effect of different types of statins on the incidence of liver cancer.

**Methods:**

Relevant articles were systematically retrieved from PubMed, EBSCO, Web of Science, and Cochrane Library databases from inception until July 2022 to explore the relationship between lipophilic statins or hydrophilic statins exposure and the incidence of liver cancer. The main outcome was the incidence of liver cancer.

**Results:**

Eleven articles were included in this meta-analysis. The pooled results showed a reduced incidence of liver cancer in patients exposed to lipophilic statins (OR=0.54, *p <* 0.001) and hydrophilic statins (OR=0.56, *p <* 0.001) compared with the non-exposed cohort. Subgroup analysis showed that both exposures to lipophilic (Eastern countries: OR=0.51, *p <* 0.001; Western countries: OR=0.59, *p <* 0.001) and hydrophilic (Eastern countries: OR=0.51, *p <* 0.001; Western countries: OR=0.66, *p*=0.019) statins reduced the incidence of liver cancer in Eastern and Western countries, and the reduction was most significant in Eastern countries. Moreover, atorvastatin (OR=0.55, *p <* 0.001), simvastatin (OR=0.59, *p <* 0.001), lovastatin (OR=0.51, *p <* 0.001), pitavastatin (OR=0.36, *p*=0.008) and rosuvastatin (OR=0.60, *p*=0.027) could effectively reduce the incidence of liver cancer, unlike fluvastatin, cerivastatin and pravastatin.

**Conclusion:**

Both lipophilic and hydrophilic statins contribute to the prevention of liver cancer. Moreover, the efficacy was influenced by the region and the specific type of statins used.

## 1 INTRODUCTION

Liver cancer is the sixth leading cancer and the third major cause of cancer death worldwide, with the highest incidence and mortality observed in East Asian countries [1, 2]. Although the incidence of liver cancer has decreased in recent years, the incidence still reached 4.7% in 2020 [2]. Hepatocellular carcinoma (HCC) is the most common form of liver cancer, and the incidence factors mainly include hepatitis virus infection, aflatoxin exposure, high alcohol intake, and so on [1]. Indeed, the influencing factors may be subject to significant heterogeneity due to differences in regions, with hepatitis B virus (HBV) infection predominating in most Asian and African countries, hepatitis C virus (HCV) in Western Europe and North America, while Central and Eastern Europe most affected by alcohol intake [1]. Surgery remains the mainstay of curative treatment for liver cancer [3]. However, since the diagnosis is often made in the middle and late stages of disease development, the prognosis of liver cancer is dismal, with a 5-30% 5-year survival rate globally [4], highlighting the necessity to explore new approaches to prevent liver cancer.

Statins represent a competitive class of 3-hydroxy-3-methylglutaryl coenzyme-A (HMG-CoA) reductase (HMGCR) inhibitors with pleiotropic anticancer properties in addition to excellent hypocholesterolemic effects [5, 6]. An increasing body of evidence suggests that statins exhibit preventive effects against gastric cancer, colorectal cancer, breast cancer, prostate cancer, and other cancer types [7-10]. Besides, the anticancer effect has been explored in liver cancer. Previous meta-analyses found that the use of statins was associated with a reduced risk of liver cancer in a dose-dependent manner [11, 12]. Depending on the solubility in water or lipid-containing media, statins can be divided into lipophilic statins (atorvastatin, fluvastatin, lovastatin, simvastatin, pitavastatin, cerivastatin) and hydrophilic statins (pravastatin, rosuvastatin) [13]. Both statins possess lipid-lowering and anticancer effects, which may vary in metabolic pathways, anticancer mechanisms, and other aspects [14]. There was a rich literature substantiating that lipophilic and hydrophilic statins exerted different effects in preventing gastric cancer, colorectal cancer, prostate cancer, etc [7, 15, 16]. Although previous meta-analyses have assessed the preventive effects of statins against liver cancer, inconsistent results have been reported [11, 17, 18]. Accordingly, there is a need to explore the differences between lipophilic and hydrophilic statins in preventing liver cancer. This study aims to explore the correlation between lipophilic and hydrophilic statins exposure and the incidence of liver cancer.

## 2 MATERIALS AND METHODS

### 2.1 Search Strategy

This meta-analysis systematically investigated studies that assessed the effect of exposure to lipophilic statins or hydrophilic statins on the incidence of liver cancer. The following search terms were used to search PubMed, EBSCO, Web of Science, and the Cochrane Library databases until July 2022 as recommended by the Preferred Reporting Items for Systematic Reviews and Meta-Analyses (PRISMA) statement [19]: (statin OR statins OR atorvastatin OR rosuvastatin OR lipitor OR crestor OR lovastatin OR simvastatin OR pravastatin OR fluvastatin OR pitavastatin OR cerivastatin) AND (hepatocellular carcinoma OR liver cancer OR hepatoma OR HCC). The references of the selected articles were also searched manually to ensure potentially relevant articles were not missed.

### 2.2 Study Selection

Articles that explored the association between lipophilic statins/hydrophilic statins exposure and liver cancer incidence were analyzed in this meta-analysis. If duplicate reports existed, the most recent or more comprehensive one was selected.

#### 2.2.1 Inclusion Criteria

(1) Randomized controlled trials (RCTs) or observational studies (cohort and case-control studies);

(2) Studies that explored the association between lipophilic statins or hydrophilic statins exposure and the incidence of liver cancer;

(3) Studies reported outcomes of liver cancer incidence.

#### 2.2.2 Exclusion Criteria

(1) Studies not in English;

(2) Studies not original;

(3) Experimental studies;

(4) Duplicate studies;

(5) Studies with data not extractable.

### 2.3 Data Extraction and Quality Assessment

Data extraction, including the author, year of publication, country, period, inclusion and exclusion criteria, and types of statins, was performed by two investigators independently from the included articles. Data with objections were re-extracted by a third person. The above data were analyzed as subgroups by area of study and types of statins. The RCTs were assessed using the Cochrane Collaboration tool, and observational studies were assessed by the Newcastle-Ottawa Quality Assessment Scale.

### 2.4 Statistical Analyses

The correlation between exposure to lipophilic statins or hydrophilic statins and the incidence of liver cancer was expressed using odds ratio (OR) with 95% confidence intervals (95%CI). STATA 12.0 Software was used to assess the heterogeneity by Cochrane Collaboration's risk of a tool, I^2^ statistics, and *p*-values. Heterogeneity was considered significant when I^2^ > 50%, *p* ≤ 0.05, while it was not significant when I^2^≤50%, *p* > 0.05. Given the heterogeneity in types and dosages of statins, races, and regions, the random effects model was adopted uniformly. The *p*-value for interaction <0.05 was considered statistically significant. Begg's analysis was used to assess publication bias and sensitivity analysis was carried out to evaluate the stability of the results.

## 3 RESULTS

### 3.1 Description of Studies Included in the Meta-analysis

A total of 3011 studies were retrieved from the databases and 2 from other sources. 1978 duplicate studies were excluded, and 973 were removed after screening the title and/or abstract. 51 studies were eliminated after carefully reading the full text. Finally, 11 studies were included in this meta-analysis [20-30]. More details are shown in Fig. (**1**).

### 3.2 Study Characteristics

A total of 11 articles comprising a total population of 2371626 patients assessed the relationship between lipophilic or hydrophilic statins exposure and liver cancer incidence was included in this meta-analysis. These studies were conducted in China (n=3), Korea (n=3), the United States of America (USA) (n=3), and the United Kingdom (UK) (n=2). This meta-analysis included 6 case-control studies and 6 cohort studies (one article included both a case-control study and a cohort study) but no RCT. The studies started as early as 1988 and ended as late as 2017. The inclusion criteria were mainly the diagnosis of liver cancer, and the exclusion criteria mostly included a previous cancer history. Patients included in the meta-analysis had comorbidities such as diabetes, kidney disease, hepatitis, or liver disease. The definition of statin exposure varied, according to the administration time, dose, or number of prescriptions. Most of the adjustment factors analyzed included basic conditions such as age and sex, the use of other drugs, and so on. Furthermore, more detailed characteristics are provided in Table **1** and Table **S1**.

The above observational studies were assessed using the Newcastle-Ottawa Quality Assessment Scale in terms of quality, and the results obtained met the quality requirements for meta-analysis (Table **S2**).

### 3.3 Correlation between Statins Types and Incidence of Liver Cancer

**Fig. (2):**
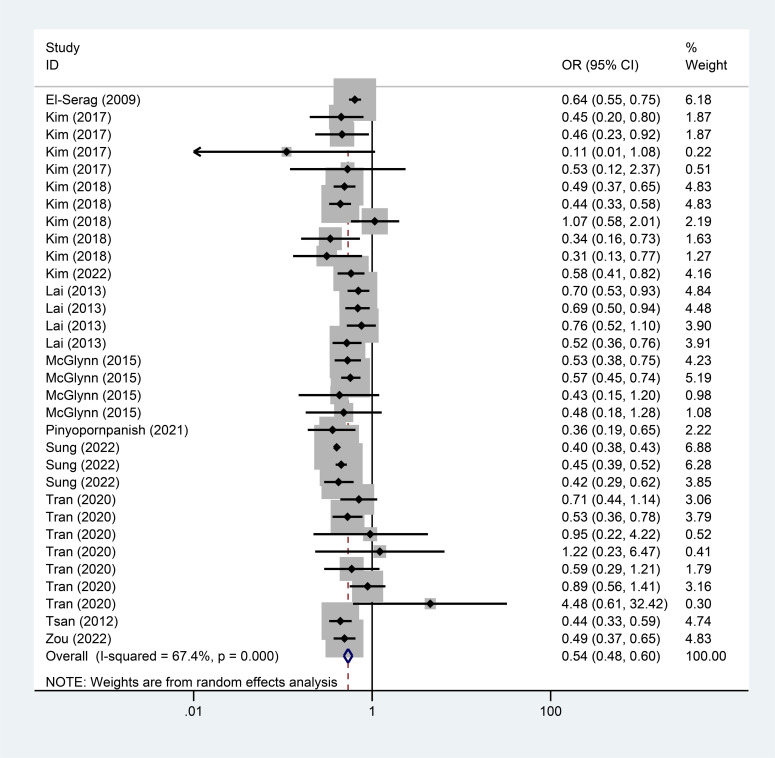
Correlation between statins types and incidence of liver cancer (Part 1).

**Fig. (3):**
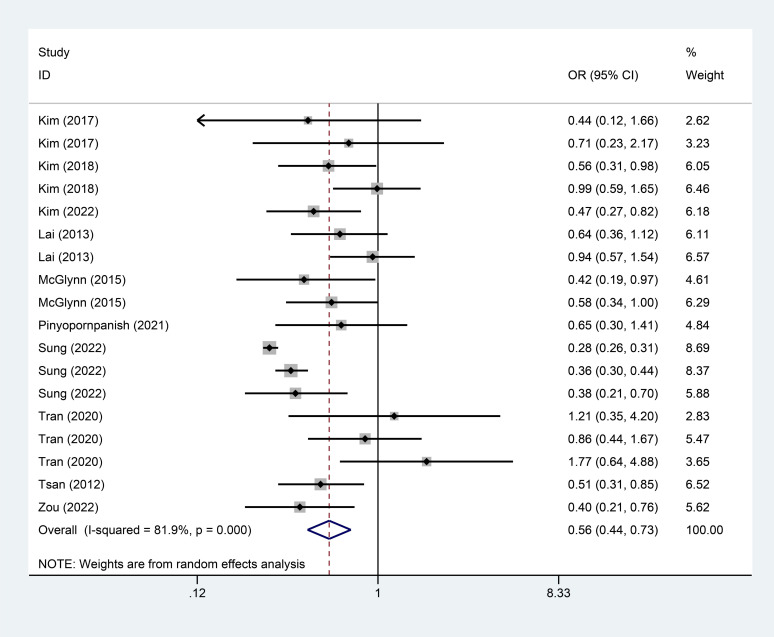
Correlation between statins types and incidence of liver cancer (Part 2).

The meta-analysis results showed that both lipophilic (OR=0.54, 95%CI=[0.48, 0.60], I^2^=67.4%, *p <* 0.001) and hydrophilic (OR=0.56, 95%CI=[0.44, 0.73], I^2^=81.9%, *p <* 0.001) statins-exposed populations experienced a significantly lower incidence of liver cancer than non-statins-exposed populations (Fig. **2**, Fig. **3**).

In addition, further subgroup analyses were performed to examine the relationship between lipophilic or hydrophilic statins exposure and the incidence of liver cancer. Firstly, a subgroup analysis was performed based on lipophilic statins use. According to the regional subgroups where the studies were conducted, in 6 studies located in Eastern countries, including China and Korea, the incidence of liver cancer was reduced in populations using lipophilic statins compared to those not using statins (OR=0.51, 95%CI=[0.45, 0.58], *p <* 0.001). Similarly, in Western countries (UK, USA), liver cancer incidence decreased in populations exposed to lipophilic statins compared to those not exposed to statins (OR=0.59, 95%CI=[0.52, 0.66], *p <* 0.001). Moreover, lipophilic statins were more effective in reducing the incidence of liver cancer in Eastern countries than in Western countries.

Stratification based on the specific statins types showed that the incidence of liver cancer was lower in the atorvastatin (OR=0.55, 95%CI=[0.46, 0.65], *p <* 0.001), simvastatin (OR=0.59, 95%CI=[0.51, 0.67], *p <* 0.001), lovastatin (OR=0.51, 95%CI=[0.38, 0.67], *p <* 0.001), and pitavastatin (OR=0.36, 95%CI=[0.17, 0.77], *p*=0.008) exposed groups compared to the non-statins-exposed group. However, there was no statistically significant difference between the fluvastatin *(p*=0.185) and cerivastatin (*p*=0.252) exposed groups and the non-statins-exposed group in liver cancer incidence. According to the above findings, pitavastatin yielded the best effect in reducing the incidence of liver cancer. Detailed results are shown in Table **2**.

Secondly, a subgroup analysis was conducted based on hydrophilic statins use. The geographically based subgroup analysis showed a decrease in the incidence of liver cancer in populations with hydrophilic statins use in both Eastern (OR=0.51, 95%CI=[0.38, 0.69], *p <* 0.001) and Western countries (OR=0.66, 95%CI=[0.47, 0.93], *p*=0.019). In comparison, hydrophilic statins yielded a more significant reduction in the incidence of liver cancer in Eastern countries than in Western countries.

Moreover, stratification according to the specific types of statins used showed that liver cancer incidence declined in patients treated with rosuvastatin compared non-treated statins population (OR=0.60, 95%CI=[0.39, 0.95], *p*=0.027). In contrast, pravastatin exposure did not affect liver cancer incidence *(p*=0.101). Further details are provided in Table **3**.

### 3.4 Publication Bias and Sensitivity Analysis

Begg's analysis showed no significant publication bias in studies assessing the effect of lipophilic statins exposure on liver cancer incidence (*p*=0.486) (Fig. **S1**), and the sensitivity analysis, which involved eliminating studies one by one yielded stable results (Fig. **S2**). Moreover, Begg's analysis of the effect of hydrophilic statins exposure on liver cancer incidence showed no significant publication bias (*p*=0.940) (Fig. **S3**) and the results of sensitivity analysis were stable (Fig. **S4**).

## 4 DISCUSSION

The pleiotropic anticancer properties of statins reportedly exert potential preventive effects against liver cancer [11, 12]. It has been discovered that statins can exert anticancer effects by inducing apoptosis in liver cancer cells and inhibiting cell cycle progression [31, 32]. Statins inhibit GTPase activity by inhibiting HMG-CoA reductase in the liver, blocking the phosphorylation and activation of MYC oncogene [33]. Then the inactivation of MYC can lead to differentiation and dormancy of liver cancer cells, thus inducing sustained regression of liver cancer [34]. Moreover, statins can cause tumor cell cycle arrest and apoptosis by blocking the production of methylvalproic acid (MVA) and its downstream metabolites such as farnyl pyrophosphate (FPP) and germanyl diphosphate (GGPP) [35, 36]. Statins can also inhibit the proliferation of tumor cells by activating nuclear factor erythroid2-related factor 2 (Nrf-2) [37]. In addition, by inhibiting nuclear factor kappa-B (NF-κB), statins can promote apoptosis of tumor cells [38]. Overall, the above studies substantiate that statins can improve the risk of liver cancer.

The results of this meta-analysis confirmed the above inferences. First, the pooled results showed that both lipophilic and hydrophilic statins could reduce the incidence of liver cancer. Indeed, the preventive effect of statins on liver cancer may be based on the ability to inhibit tumor cell proliferation and promote its apoptosis. From the perspective of chemical structure, statins are composed of three parts: 3,5-dihydroxyheptanoic acid structural fragment similar to HMG-CoA, a complex ring structure that combines statin molecules with HMG-CoA reductase, and a side chain ring structure that determines the solubility [13]. Due to the hydroxyl group and the methanesulfonamide group on the hydrophobic ring structure, pravastatin and rosuvastatin have greater hydrophilicity compared to similar drugs [39]. The hydrophilicity or lipophilicity of statins, determined by their chemical structure, affects the absorption of drugs. Hydrophilic statins can be rapidly dissolved in the gastrointestinal tract, mainly through active transport into hepatocytes, while lipophilic statins are more likely to be passively diffused and absorbed through the membrane [40]. The greater hepatoselectivity of hydrophilic statins rendered them easier to stay in the liver and play a role, while the superior ability to cross and interact with cell membranes meant that lipophilic statins were more powerful in lowering cholesterol and fighting cancer. However, previous meta-analyses mostly concluded that lipophilic statins exposure could improve the incidence of liver cancer to some extent while hydrophilic statins could not, or both could or even neither could, which was not consistent with the findings of this study [11, 17, 18]. This discrepancy may be due to the relatively small number of studies and their limited sample size.

Moreover, this meta-analysis revealed that both lipophilic and hydrophilic statins were more effective in preventing liver cancer in Eastern countries than in Western countries. One of the possible reasons is the difference in the population base of liver cancer. The incidence rate of liver cancer in Eastern countries is higher than that in Western countries, and the large population of liver cancer patients in Eastern countries may also be one of the reasons for this discrepancy [1, 2]. It may also be related to the different pathogenic factors in different regions. In Eastern countries, HBV infection is the predominant factor in the occurrence of liver cancer, while HCV infection, alcohol intake, and fatty liver are predominant in Western countries [1]. It has been reported that statins can disrupt HCV RNA replication by inhibiting protein geranylgeranylation [41]. Statins can also inhibit the epidermal growth factor (EGF) signaling pathway, which increases the entry of HCV cells [42, 43]. Although some meta-analyses found that statins could reduce the incidence of HCC in patients with chronic hepatitis B, experimental studies correlating statins with HBV were lacking [44, 45]. A fatty liver results from excessive fat deposition in liver cells [46]. Statins, as a lipid-lowering drug, can effectively reduce the cholesterol levels in the liver. Some meta-analyses substantiated the protective effect of statins on nonalcoholic fatty liver disease (NAFLD) [47, 48]. However, there is a lack of research on the relationship between statins and alcohol-related liver disease. The differences between Eastern and Western countries are worth exploring to prevent liver cancer with statins.

After stratification according to specific statins types, we found that atorvastatin, simvastatin, lovastatin, pitavastatin, and rosuvastatin could effectively prevent liver cancer, but fluvastatin, cerivastatin, and pravastatin were ineffective, consistent with the literature. Atorvastatin is found to inhibit hepatic stellate cell (HSC) activation and fibrosis and eliminate oxidative stress to all deviating liver cancer progression [49, 50]. Atorvastatin can also adjust the clinical prognosis of liver signature (PLS), predicting the risk of HCC in patients with liver disease [51]. A study shows that nano delivery of simvastatin can target liver sinusoidal endothelial cells to remodel the tumor microenvironment for HCC [52]. Besides, simvastatin can induce apoptosis of liver cancer cells and impair cell cycle progression [32]. And simvastatin can also reduce the occurrence and metastasis of tumors by reducing the senescence-associated secretory phenotype (SASP) [53]. A study shows that lovastatin can destroy HCV RNA replication by affecting host protein geranylation, which may impact the progression of liver cancer with hepatitis C onset [41]. Moreover, lovastatin has been shown to inhibit the activity of liver cancer cells [54]. Nonetheless, basic research on the effects of other statins on liver cancer is scarce. Besides, the role of some statins in preventing other cancers has been confirmed by basic experiments. Lovastatin can inhibit proliferation and promote apoptosis of breast cancer cells [54]. Rosuvastatin can prevent prostate cancer by inhibiting cell proliferation and spheroid formation of prostate cancer PC-3 cells and inhibiting the epithelial-mesenchymal transition (EMT) [55]. In our meta-analysis, pitavastatin yielded the best effect. Consistently, a study finds that statins, especially pitavastatin, yield the highest killing rate against the cells with PTEN mutation of the genetically modified cancer gene [56]. However, due to the limited clinical application time of pitavastatin, there was a lack of relevant basic research to support this conclusion.

This study conducted a comprehensive meta-analysis of the association between statin types and liver cancer incidence. Importantly, we provided compelling evidence of the preventive effects of lipophilic and hydrophilic statins on liver cancer, and the anticancer effects of lipophilic and hydrophilic statins were affected by region and specific types. Unfortunately, some limitations were found in this study. First, the number of included clinical studies was limited, especially the lack of RCTs. Moreover, the inconsistent influencing factors and treatment of liver cancer in the data of the included studies may be sources of heterogeneity.

## CONCLUSION

In conclusion, lipophilic and hydrophilic statins could reduce the risk of liver cancer. Compared with Western countries, lipophilic statins and hydrophilic statins exerted good effects on the incidence of liver cancer in Eastern countries. Moreover, atorvastatin, simvastatin, lovastatin, pitavastatin, and rosuvastatin could prevent liver cancer, while fluvastatin, cerivastatin, and pravastatin could not.

## AUTHORS’ CONTRIBUTIONS

All authors contributed to the study's conception and design. Material preparation was performed by Xueqiang Ma. Data collection and analysis were performed by Xingfen Zhang and Dandi Lou. The first draft of the manuscript was written by Rongrong Fu, Feng Wu, and Dingcheng Zheng, and all authors commented on previous versions of the manuscript. All authors read and approved the final manuscript.

## Figures and Tables

**Fig. (1):**
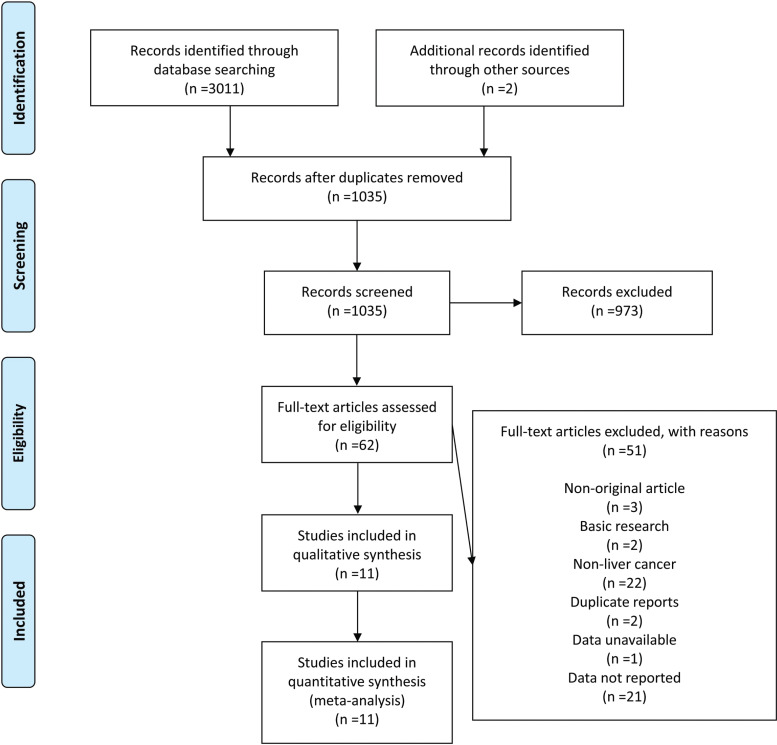
PRISMA Flowdiagram.

**Table 1:** Characteristics of included studies in the meta-analysis of the association between statins types and incidence of liver cancer.

**Author, Year **	**Country**	**Period**	**Inclusion Criteria**	**Exclusion Criteria**	**Administration Time / Dose of Statins**	**Types of Statins**	**Research Type**
El-Serag, 2009	USA	2001.1.1-2002.12.31	Diagnosis of HCC and diabetes	Without any VA pharmacy use	-	Simvastatin	case-control study
Kim, 2017	Korea	2002-2013	Diagnosis of T2DM; diagnosis of HCC with at least a prior 5-year HCC-free period; no diagnosis of other cancers; no prior diagnosis of any cancer	No supporting clinical codes indicating the presence of HCC	-	Atorvastatin, Simvastatin, Lovastatin, Pitavastatin, Rosuvastatin, Pravastatin	case-control study
Kim, 2018	Korea	2002-2013	Diagnosis of HCC with at least a three-year cancer free period; no prior diagnosis of any cancer	No supporting clinical codes indicating the presence of HCC	≥30 days	Atorvastatin, Simvastatin, Fluvastatin, Lovastatin, Pitavastatin, Rosuvastatin, Pravastatin	case-control study
Kim, 2022	Korea	2009.1.1-2017.12.31	Administration of dialysis for at least 3 months; diagnosis of mono-infection with either HBV or HCV	History of cancer or organ transplantation prior to dialysis initiation	≥28 cDDDs	-	cohort study
Lai, 2013	China	2000-2009	Diagnosed HCC	Prior diagnosis of HCC or any other cancer	-	Atorvastatin, Simvastatin, Fluvastatin, Lovastatin, Rosuvastatin, Pravastatin	case-control study
McGlynn, 2015	UK	1988-2011	Diagnosis of liver cancer; no prior diagnosis of other cancers	Liver metastases	≥2 prescriptions	Atorvastatin, Simvastatin, Fluvastatin, Cerivastatin, Rosuvastatin, Pravastatin	case-control study
Pinyopornpanish, 2021	USA	2002.7-2016.6	Diagnosis of cirrhosis	Prior diagnosis of HCC; history of excessive alcohol consumption; concurrent diagnosis of other liver diseases; unknown date of cirrhosis diagnosis or liver biopsy; follow-up of less than 6 months or HCC diagnosed within 6 months of cirrhosis diagnosis; insufficient baseline clinical and laboratory data	-	-	cohort study
Sung, 2022	China	1999–2015	History of statins therapy for at least 90 days	History of ESRD or CKD for less than 90 days, HIV, or kidney transplant; deceased at the baseline; history of liver cancer; ESRD patients without dialysis information	≥90 days	-	cohort study
Tran, 2020	UK	1999.1.1-2011.4.30	Diagnosis of liver cancer	-	-	Atorvastatin, Simvastatin, Fluvastatin, Cerivastatin, Rosuvastatin, Pravastatin	case-control study
Tran, 2020	UK	2006-2010	Diagnosis of liver cancer	Prior diagnosis of any cancer	-	Atorvastatin, Simvastatin, Fluvastatin, Rosuvastatin	cohort study
Tsan, 2012	China	1997-2008	Diagnosis of HBV infection without HCV infection	Prior diagnosis of HCC	≥28 cDDDs	-	cohort study
Zou, 2022	USA	2003.1-2009.6	Diagnosed with NAFLD	More than 2 enrollments in Optum and more than 6 months apart between 2 enrollments; diagnosis of HBV, HCV, other liver diseases, and any cancer other than HCC; statins exposure before NAFLD diagnosis; prior diagnosis of HCC; died or were lost to follow-up less than 6 months	≥30 cDDDs	-	cohort study

**Abbreviations:** USA, United States of America; HCC, hepatocellular carcinoma; VA, Veterans Affairs; HCV, hepatitis C virus; HBV, hepatitis B virus; T2DM, type 2 diabetes; cDDD, cumulative defned daily dose; UK, United Kingdom; ESRD, end stage of real disease; CKD, chronic kidney disease; HIV, human immunodeficiency virus; NAFLD, non-alcoholic fatty liver disease; -, data unavailable.

**Table 2:** Statistical results of the association between lipophilic statins exposure and incidence of liver cancer.

**Subgroup**	**No. of Studies**	**OR**	**95%CI**	***p***	**I^2^ (%)**
**Area**					
Eastern countries	6	0.51	[0.45, 0.58]	<0.001	65.8
Western countries	6	0.59	[0.52, 0.66]	<0.001	14.6
**Statins type**					
Atorvastatin	7	0.55	[0.46, 0.65]	<0.001	25.8
Simvastatin	8	0.59	[0.51, 0.67]	<0.001	31.5
Fluvastatin	6	0.72	[0.45, 1.17]	0.185	48.9
Lovastatin	4	0.51	[0.38, 0.67]	<0.001	9.3
Pitavastatin	2	0.36	[0.17, 0.77]	0.008	0.0
Cerivastatin	2	0.61	[0.26, 1.42]	0.252	0.0

**Abbreviations:** No., number; OR, odds ratio; 95%CI: 95% confidence intervals.

**Table 3:** Statistical results of the association between hydrophilic statins exposure and incidence of liver cancer.

**Subgroup**	**No. of Studies**	**OR**	**95%CI**	***p***	**I^2^ (%)**
**Area**					
Eastern countries	6	0.51	[0.38, 0.69]	<0.001	84.3
Western countries	5	0.66	[0.47, 0.93]	0.019	32.0
**Statins Type**					
Rosuvastatin	7	0.60	[0.39, 0.95]	0.027	42.7
Pravastatin	6	0.82	[0.65, 1.04]	0.101	0.0

**Abbreviations:** No., number; OR, odds ratio; 95%CI: 95% confidence intervals.

## Data Availability

The data and supportive information are available within the article.

## LIST OF ABBREVIATIONS

HCC: Hepatocellular carcinoma
HBV: Hepatitis B virus
HCV: Hepatitis C virus
HMG-CoA: 3-hydroxy-3-methylglutaryl coenzyme-A
HMGCR: 3-hydroxy-3-methylglutaryl coenzyme-A reductase
PRISMA: Preferred reporting items for systematic reviews and meta-analyses
RCT: Randomized controlled trials
OR: Odds ratio
95%CI: 95% confidence intervals
USA: United States of America
UK: United Kingdom
MVA: Methylvalproic acid
FPP: Farnyl pyrophosphate
GGPP: Germanyl diphosphate
Nrf-2: Nuclear factor erythroid2-related factor 2
NF- κB: Nuclear factor kappa-B
EGF: Epidermal growth factor
NAFLD: Nonalcoholic fatty liver disease
HSC: Hepatic stellate cells
PLS: Prognosis of liversignature
SASP: Senescence-associated secretory phenotype
EMT: Epithelial-mesenchymal transition

## STANDARDS OF REPORTING

PRISMA guidelines and methodology were followed.

PRISMA checklist is available on the publisher’s website

## SUPPORTIVE/SUPPLEMENTARY MATERIAL

PRISMA checklist is available as supplementary material on the publisher’s website along with the published article. SFig. **1.** Publication bias of the association between lipophilic statins exposure and incidence of liver cancer (Begg's, *p*=0.486). SFig. **2.** Sensitivity analysis of the association between lipophilic statins exposure and incidence of liver cancer. SFig. **3.** Publication bias of the association between hydrophilic statins exposure and incidence of liver cancer (Begg's, *p*=0.940). SFig. **4.** Sensitivity analysis of the association between hydrophilic statins exposure and incidence of liver cancer. Table **S1**. Characteristics of included studies in the meta-analysis of the association between statin types and incidence of liver cancer. Table **S2**. Quality assessment of studies included.
